# G‐alpha interacting protein interacting protein, C terminus 1 regulates epileptogenesis by increasing the expression of metabotropic glutamate receptor 7

**DOI:** 10.1111/cns.13746

**Published:** 2021-10-22

**Authors:** Yong Liu, You Wang, Juan Yang, Tao Xu, Changhong Tan, Peng Zhang, Qiankun Liu, Yangmei Chen

**Affiliations:** ^1^ Department of Neurology The Second Affiliated Hospital of Chongqing Medical University Chonqing China; ^2^ Department of Neurology The Affiliated Hospital of Zunyi Medical University Guizhou China

**Keywords:** epilepsy, G‐alpha interacting protein interacting protein, C terminus 1, metabotropic glutamate receptor 7, protein transport, synaptic transmission

## Abstract

**Aims:**

It has been reported that the G‐alpha interacting protein (GAIP) interacting protein, C terminus 1 (GIPC1/GIPC) engages in vesicular trafficking, receptor transport and expression, and endocytosis. However, its role in epilepsy is unclear. Therefore, in this study, we aimed to explore the role of GIPC1 in epilepsy and its possible underlying mechanism.

**Methods:**

The expression patterns of GIPC1 in patients with temporal lobe epilepsy (TLE) and in mice with kainic acid (KA)‐induced epilepsy were detected. Behavioral video monitoring and hippocampal local field potential (LFP) recordings were carried out to determine the role of GIPC1 in epileptogenesis after overexpression of GIPC1. Coimmunoprecipitation (Co‐IP) assay and high‐resolution immunofluorescence staining were conducted to investigate the relationship between GIPC1 and metabotropic glutamate receptor 7 (mGluR7). In addition, the expression of mGluR7 after overexpression of GIPC1 was measured, and behavioral video monitoring and LFP recordings after antagonism of mGluR7 were performed to explore the possible mechanism mediated by GIPC1.

**Results:**

GIPC1 was downregulated in the brain tissues of patients with TLE and mice with KA‐induced epilepsy. After overexpression of GIPC1, prolonged latency period, decreased epileptic seizures and reduced seizure severity in behavioral analyses, and fewer and shorter abnormal brain discharges in LFP recordings of KA‐induced epileptic mice were observed. The result of the Co‐IP assay showed the interaction between GIPC1 and mGluR7, and the high‐resolution immunofluorescence staining also showed the colocalization of these two proteins. Additionally, along with GIPC1 overexpression, the total and cell membrane expression levels of mGluR7 were also increased. And after antagonism of mGluR7, increased epileptic seizures and aggravated seizure severity in behavioral analyses and more and longer abnormal brain discharges in LFP recordings were observed.

**Conclusion:**

GIPC1 regulates epileptogenesis by interacting with mGluR7 and increasing its expression.

## 1 INTRODUCTION

Epilepsy is a common, chronic, and serious neurological disorder that affects approximately 65 million people worldwide.^1^ However, more than one‐third of patients remain uncontrolled after treatment with anti‐seizure medications (ASMs).^2^, ^3^ Numerous studies have identified various pathological mechanisms of epilepsy, including neuroinflammation,^4^ synaptic transmission,^5^, ^6^ metabolic disorders,^7^ and an impaired blood‐brain barrier.^4^ However, among these mechanisms, an imbalance between excitatory and inhibitory synaptic transmission is known as the common epileptogenic mechanism.^8^, ^9^, ^10^ Therefore, further studies on the role of synaptic transmission in epileptogenesis are important to develop more effective epilepsy treatments.

G‐alpha interacting protein (GAIP) is a regulator of G protein signaling. Initially, it was found that the GAIP interacting protein, C terminus 1 (GIPC1/GIPC) contains a PSD‐95/Dlg/ZO‐1 (PDZ) structural domain that binds to the GAIP C terminus; hence, it acquired the name GIPC protein.^11^ GIPC1 is the main member of the GIPC protein family that is relatively highly expressed in brain tissue.^12^ As a PDZ domain‐containing protein, GIPC1 can bind to a group of proteins that possess a PDZ‐binding motif (PBM) via its PDZ domain. To date, it has been reported that there are more than 50 protein‐binding partners of GIPC1, most of which play essential roles in neuronal development.^13^ Hence, GIPC1 may play a multifaceted role in the central nervous system (CNS). In addition, it has been reported that gene mutations in the GIPC protein family may be associated with the development of myoclonus, primary tremor, and juvenile epilepsy.^14^ Furthermore, previous studies have indicated that many scaffolding proteins^15^, ^16^, ^17^ are involved in epilepsy; hence, we speculate that GIPC1, a scaffolding protein, also participates in epilepsy. Through binding to various proteins, GIPC1 plays a role in many biological processes such as vesicular trafficking,^13^, ^18^ protein transport and expression,^19^, ^20^, ^21^ endocytosis,^22^ signal transduction,^23^, ^24^ and tumorigenesis.^11^ Therefore, GIPC1 plays an important role in synaptic transmission. Given the importance of synaptic transmission in epileptogenesis, we speculate that GIPC1 is involved in epileptogenesis by regulating synaptic transmission.

Therefore, in the present study, we explored the expression patterns of GIPC1 in the brain tissues of humans and mice, and then investigated the effects of GIPC1 on seizure susceptibility and brain electrical activity in kainic acid (KA)‐induced epileptic mice. Moreover, we further elucidated the underlying mechanism mediated by GIPC1 in regulating epileptic activities.

## 2 METHODS

### 2.1 Human brain tissue

This research was conducted in accordance with the Declaration of Helsinki and the ethical principles of the National Institutes of Health. This research was also approved by the Ethics Committee of the Second Affiliated Hospital of Chongqing Medical University. All patients and their guardians were informed of the use of clinical data and brain tissues and provided informed consent. All temporal neocortex samples were obtained from the Xinqiao Hospital of Third Military Medical University and the First Affiliated Hospital of Chongqing Medical University. With the diagnostic criteria proposed by the International League Against Epilepsy (ILAE),^25^ the temporal neocortex samples from 15 refractory temporal lobe epilepsy (TLE) patients were enrolled in the epilepsy group. Meanwhile, the control samples were obtained from 14 traumatic brain injury patients who underwent craniotomy due to an increased intracranial pressure. None of the patients in the control group had a history of epilepsy, seizures, or any other neurological diseases and were exposed to ASMs. The clinical characteristics of TLE patients and control patients are listed in Table 1 and Table 2, respectively.

**TABLE 1:** Clinical features of TLE patients

No.	Sex	Age (years)	Course (years)	ASMs	Resected tissue	Pathology diagnosis
1	F	13	5	CBZ, PHT, LTG	LTN	G, NL
2	M	17	3	CBZ, VPA, PHT,	LTN	G, NL
3	M	20	7	VPA, PB, CBZ, LEV	LTN	G, NL, ND
4	F	8	6	OXC, CLZ, VPA, TPM	RTN	G
5	F	10	5	CBZ, VPA, TPM	RTN	G, NL, ND
6	M	19	9	OXC, VPA, GBP	LTN	G, NL, ND
7	M	23	7	VPA, LEV, PHT	LTN	G, NL
8	F	25	13	LTG, TPM, CBZ	RTN	G
9	F	7	4	OXC, LEV, PB	LTN	G, NL
10	M	32	15	CBZ, PHT, LTG	RTN	G, NL, ND
11	M	23	11	CBZ, VPA, CLZ, TPM	RTN	G, NL, ND
12	F	18	9	VPA, PB, CBZ, LEV	LTN	G, NL, ND
13	M	37	15	PHT, TPM, PB	RTN	G
14	F	29	19	CBZ, VPA, LEV, PB	LTN	G, NL
15	M	31	13	OXC, PHT, LTG	LTN	G, NL, ND

Abbreviations: ASMs, anti‐seizure medications; CBZ, carbamazepine; CLZ, clonazepam; F, female; G, gliosis; GBP, gabapentin; LEV, levetiracetam; LTG, lamotrigine; LTN, left temporal neocortex; M, male; ND, neuronal degeneration; NL, neuronal loss; OXC, oxcarbazepine; PB, phenobarbitone; PHT, phenytoin; RTN, right temporal neocortex; TPM, topiramate; VPA, valproic acid.

**TABLE 2:** Clinical features of control patients

No.	Sex	Age (years)	Disease	Resected tissue	Pathology diagnosis
1	F	23	Brain trauma	RTN	Normal
2	M	28	Brain trauma	LTN	Normal
3	F	15	Brain trauma	RTN	Normal
4	F	17	Brain trauma	LTN	Normal
5	M	20	Brain trauma	LTN	Normal
6	F	32	Brain trauma	RTN	Normal
7	M	19	Brain trauma	RTN	Normal
8	F	27	Brain trauma	RTN	Normal
9	F	31	Brain trauma	LTN	Normal
10	M	37	Brain trauma	RTN	Normal
11	M	26	Brain trauma	LTN	Normal
12	F	23	Brain trauma	RTN	Normal
13	M	19	Brain trauma	RTN	Normal
14	F	24	Brain trauma	LTN	Normal

Abbreviations: F, female; LTN, left temporal neocortex; M, male; RTN, right temporal neocortex.

### 2.2 Animals

All animal studies conducted in this research were approved by the Committee on Animal Research of Chongqing Medical University. All animal experiments complied with the National Institutes of Health Guide for the Care and Use of Laboratory Animals and the rules of the Animal Ethical Committee of Chongqing Medical University. The animal data reporting followed the ARRIVE 2.0 guidelines.^26^ Healthy adult male C57BL/6 mice (8−10 weeks and 20−25 g) were obtained from the Experimental Animal Center of Chongqing Medical University. All mice were kept in a specific pathogen‐free (SPF) animal facility at constant temperature (21−22℃) and humidity (50%−60%) with a 12h/12h light/dark cycle. Food and water were available freely.

### 2.3 KA‐induced epilepsy model

The procedures for intrahippocampal administration of KA were reported in previous studies.^27^, ^28^ Mice were anesthetized by intraperitioneal injection of sodium pentobarbital (50 mg/kg) and placed on a stereotaxic apparatus (RWD Life Science). Then, using a 0.5 μl syringe (Hamilton), KA (50 nl of a 20 mM solution) (Sigma‐Aldrich Co.) was injected into the right hippocampal CA1 region (anterior‐posterior (AP), 2.0 mm; medial‐lateral (MEL), 1.5 mm; dorsal‐ventral (DV), 1.4 mm) for 3 min. The syringe was left in place for 5 min and finally withdrawn slowly to minimize reflux. The mice in the control group were injected with an equal volume of 0.9% saline in the same manner.

### 2.4 Adeno‐associated virus vector construction and injection

The Adeno‐associated virus (AAV) products used in this study were constructed by Hanbio Biotechnology. The titer of the AAV vectors was 1.8 × 10^12^ vg/ml. Mice were deeply anesthetized and fixed on a stereotaxic apparatus. One microliter of the AAV vectors was bilaterally injected into the hippocampal CA1 region on one side via a 5 μl syringe at a speed of 0.2 μl/min. Then, the syringe was kept in place for 8 min and finally withdrawn slowly to prevent reflux. AAV vectors expressing the coding sequence of GIPC1 with 3× flags at its C terminus were used to overexpress GIPC1 and injected into the mice from the GIPC1 group. Meanwhile, AAV vectors that only expressed the green fluorescent protein (GFP) were injected into the mice in the Con‐GIPC1 group as a negative control. Mice in the control group were injected with an equal volume of 0.9% saline in the same manner.

### 2.5 Drug administration in vivo

On the third day after KA injection following AAV vector injection, VU6012962 (3 mg/kg, HY‐114430; MedChem Express), a highly selective negative allosteric modulator (NAM) of metabotropic glutamate receptor 7 (mGluR7), was intraperitoneally injected into mice for 7 consecutive days to inhibit mGluR7 function.^29^, ^30^ VU6012962 was dissolved in dimethylsulfoxide, and corn oil was used to aid solubility. Therefore, the vehicle groups were intraperitoneally injected with an equal volume of dimethylsulfoxide solubilized with corn oil.

### 2.6 Behavioral evaluation

After injection of KA, mice were monitored for one month by a video monitoring system (24 h per day). According to Racine's criteria (stages 0–5),^31^ spontaneous recurrent seizures (SRSs) were classified. Only mice with stage 4−5 SRSs were selected for inclusion in the epilepsy group. In addition, hippocampal local field potential (LFP) recordings were also collected to confirm the successful establishment of the KA‐induced epilepsy model.

After KA injection following AAV vector injection (3 weeks after AAV vector injection), video recordings were also used to assess the behavioral changes of the mice from the KA, Con‐GIPC1 + KA, and GIPC1 + KA groups. Similarly, after consecutive administration of VU6012962 for a week, behavioral tests were conducted.

### 2.7 Hippocampal LFP recordings

Following the behavioral assessment, hippocampal LFPs of mice were recorded. Mice were anesthetized and mounted on a stereotaxic apparatus. Then, a U‐shaped frame was used to hold the head by fixing to the skull.^32^ Prior to the recording, electrodes were implanted into the right hippocampal CA1 region and connected with the signal connector, which was fixed to the mouse skull by dental acrylic cement. The LFP was monitored and recorded with a MAP data acquisition system (Plexon) and then analyzed with Neuroexplorer software (Nex Technologies).^32^ Only clusters of spontaneous paroxysmal discharges with a frequency of above 1 Hz, an amplitude two times greater than that at baseline and a duration of at least 5 s, were defined as seizure‐like events (SLEs).^29^, ^33^

At week 4 post‐KA injection following AAV vector injection (7 weeks after AAV vector injection), LFP recordings were used to analyze abnormal discharges of the mice from the KA, Con‐GIPC1 + KA, and GIPC1 + KA groups. Similarly, at week 4 post‐KA injection following AAV vector injection, LFP recordings were conducted in the antagonism of mGluR7 experiments.

### 2.8 Western blotting

Human and mouse brain tissues were used for Western blotting analysis. Total proteins were separated with a whole protein extraction kit (Beyotime). Cell membrane proteins were extracted by the Minute™ Plasma Membrane Protein Isolation and Cell Fractionation Kit (Invent). The protein concentration was measured using the enhanced bicinchoninic acid (BCA) Protein Concentration Assay Kit (Beyotime). Proteins (20−30 µg) were loaded in each lane of a gel, separated by SDS‐PAGE, and then transferred to polyvinylidene fluoride (PVDF) membranes (Merck Millipore). After blocking with 5% skim milk, the PVDF membranes were incubated with the following primary antibodies overnight at 4℃: anti‐GIPC1 rabbit polyclonal antibody (1:2000; 14822‐1‐AP; Proteintech), anti‐mGluR7 rabbit polyclonal antibody (1:2000; CY1080; Abways), anti‐GAPDH rabbit polyclonal antibody (1:10000; 10494‐1‐AP; Proteintech), and anti‐Na^+^‐K^+^‐ATPase rabbit monoclonal antibody (1:50000; ab76020; Abcam). GAPDH and Na^+^‐K^+^‐ATPase were used as the loading controls for total protein and cell membrane proteins, respectively. On the second day, the membranes were incubated with a horseradish peroxidase‐conjugated goat anti‐rabbit IgG antibody (1:4000; SA00001‐2; Proteintech) for 1 h at room temperature, and proteins on the membranes were visualized via an enhanced chemiluminescence substrate kit (Beyotime) on a fusion imaging system (Vilber Lourmat, France). Full unedited blot images are shown in Figure S1.

### 2.9 Immunofluorescence labeling

The procedure for double‐labeled immunofluorescence staining was described previously.^34^ Human and mouse brain tissues were fixed with 4% paraformaldehyde for 24 h and then successively immersed in 20% and 30% sucrose solutions for 24 h for dehydration and cut into 16 μm frozen slices. The slices were permeabilized with 0.4% Triton X‐100 for 20 min at 37℃ and heated in a microwave oven for 20 min at 98℃ with 10 mM sodium citrate buffer. After washing, the slices were subsequently blocked with 5% goat serum (Boster) for 1 h at 37℃. Then, the slices were incubated with a mixture of the following primary antibodies overnight at 4℃: anti‐GIPC mouse monoclonal antibody (1:30; sc‐271822; Santa Cruz), anti‐mGluR7 rabbit polyclonal antibody (1:250; AF0171; Affinity), anti‐NeuN rabbit monoclonal antibody (1:200; ab177487; Abcam), and anti‐GFAP rabbit monoclonal antibody (1:5000; 12389; CST). The next day, the slices were incubated with a mixture of the following secondary antibodies in the dark at 37℃ for 1 h: Alexa Fluor 594‐conjugated goat anti‐rabbit IgG (1:50; 111‐585‐003; Jackson) and Alexa Fluor 488‐conjugated goat anti‐mouse IgG (1:50; 115‐545‐003; Jackson). Then, the slices were counterstained with 10% 4′,6‐diamidino‐2‐phenylindole (DAPI; Boster) for 20 min at 37℃. Finally, the slices were mounted in 50% glycerol/PBS, and the fluorescence images were captured under a laser scanning confocal microscope (Nikon).

### 2.10 Coimmunoprecipitation assay

Hippocampal tissues of KA‐induced epileptic mice were used for Coimmunoprecipitation (Co‐IP) analysis and lysed by immunoprecipitation lysis buffer. According to the manual of protein A/G magnetic beads (HY‐K0202; MedChem Express), 40 μl of protein A/G magnetic beads was incubated with 2 μl rabbit monoclonal IgG (3900; CST), 7.5 μl anti‐mGluR7 rabbit monoclonal antibody (04‐921; Sigma‐Aldrich; mGluR7 molecular weight: 115 kDa), and 5 μl anti‐GIPC1 antibody as mentioned above for 2−4 h at 4℃. Next, the antibody‐bead complex was incubated with protein lysates overnight at 4℃. The next day, washed magnetic beads were added with 1× loading buffer and denatured by a metal bath. Then, the samples were collected and analyzed by Western blotting. Full unedited blot images of Co‐IP assay are shown in Figure S1.

### 2.11 Statistical analysis

All data are normally distributed and homogeneous, with nonhomogeneous data being transformed into homogeneous data by data transformation^35^ and presented as the mean ±standard error of the mean (SEM). Normal distribution test was performed with the Shapiro‐Wilk test, and homogeneity of variance was assessed with Levene's test. Student's *t* test and Fisher's exact test were used for comparisons of age and sex in TLE patients and the controls, respectively. Comparisons between the two groups were performed using two‐tailed unpaired Student's *t* test, while comparisons between multiple groups were performed using one‐way analysis of variance (ANOVA) followed by a post hoc Bonferroni test. SPSS 20.0 (IBM) and GraphPad Prism 8.0.1 software were used for statistical analyses and graphing. Significance was set at *p* < 0.05.

## 3 RESULTS

### 3.1 No significant differences in sex and age between TLE patients and control subjects

Fifteen TLE patients (eight males and seven females) (Table 1) and 14 control subjects (six males and eight females) (Table 2) were enrolled in this study. The average age of the TLE group was 20.80 ± 2.33 years (range from 7 to 37 years), and the average age of the control group was 24.36 ± 1.67 years (range from 15 to 37 years). The differences in sex (*p* = 1.00) and age (*p* = 0.232) between these two groups were not statistically significant.

### 3.2 GIPC1 is decreased in human brain tissues

**FIGURE 1:**
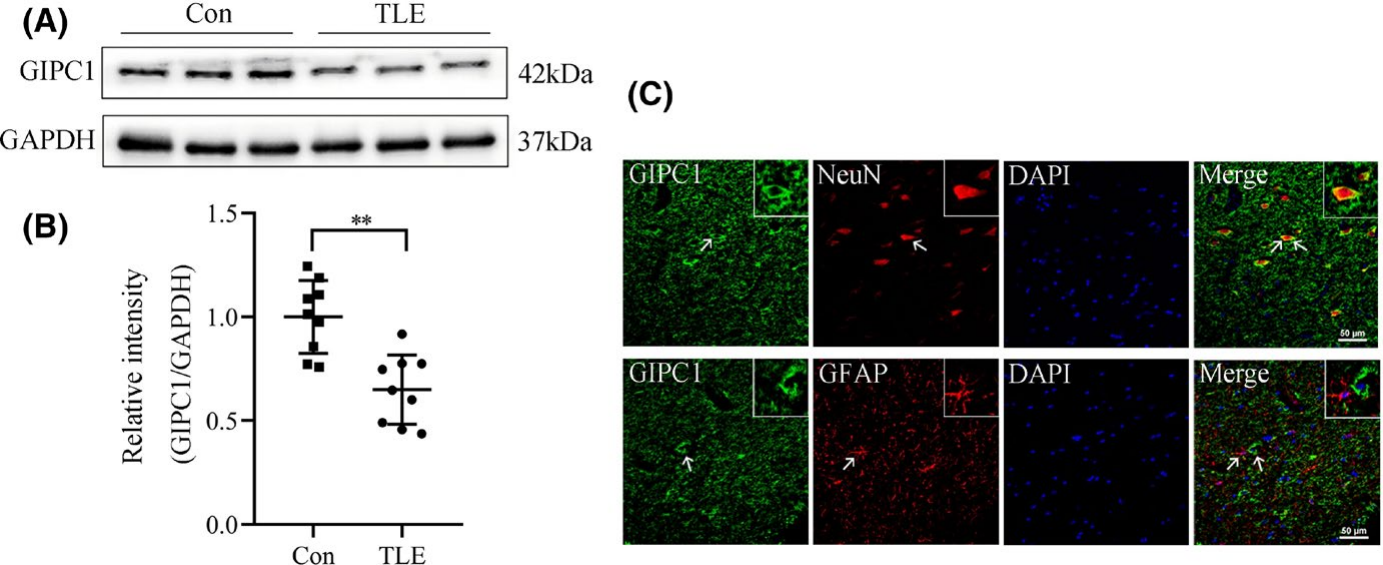
Expression and location of GAIP interacting protein, C terminus 1 (GIPC1) in TLE patients and control subjects. (A) Western blotting result of GIPC1 expression. (B) Statistical analysis indicating that the relative intensity of GIPC1/GAPDH was lower in the TLE group than that in the control group (n = 9 per group). (C) Double immunofluorescence images for GIPC1 in TLE patients. GIPC1 (green) co‐expressed (merged) with NeuN (red) but not with GFAP (red) (scale bar =50 μm). **p < 0.01 [Colour figure can be viewed at wileyonlinelibrary.com]

To determine the protein level of GIPC1 in the temporal neocortex of patients with TLE and the controls, Western blotting was carried out. As shown in Figure 1A and B, GIPC1 expression was decreased in the TLE group compared with that in the control group (*p* = 0.001). Meanwhile, we used immunofluorescence labeling to detect the cellular location of GIPC1. In the TLE group, GIPC1 (green) colocalized with the neuronal marker NeuN (red) but not with the astrocyte marker GFAP (red), indicating that GIPC1 was mainly expressed in neurons but not in astrocytes. Additionally, GIPC1 was mainly located in the cytoplasm of neurons (Figure 1C). The same results were observed in the control group (Figure S2).

### 3.3 GIPC1 is decreased in the KA‐induced epilepsy model

**FIGURE 2:**
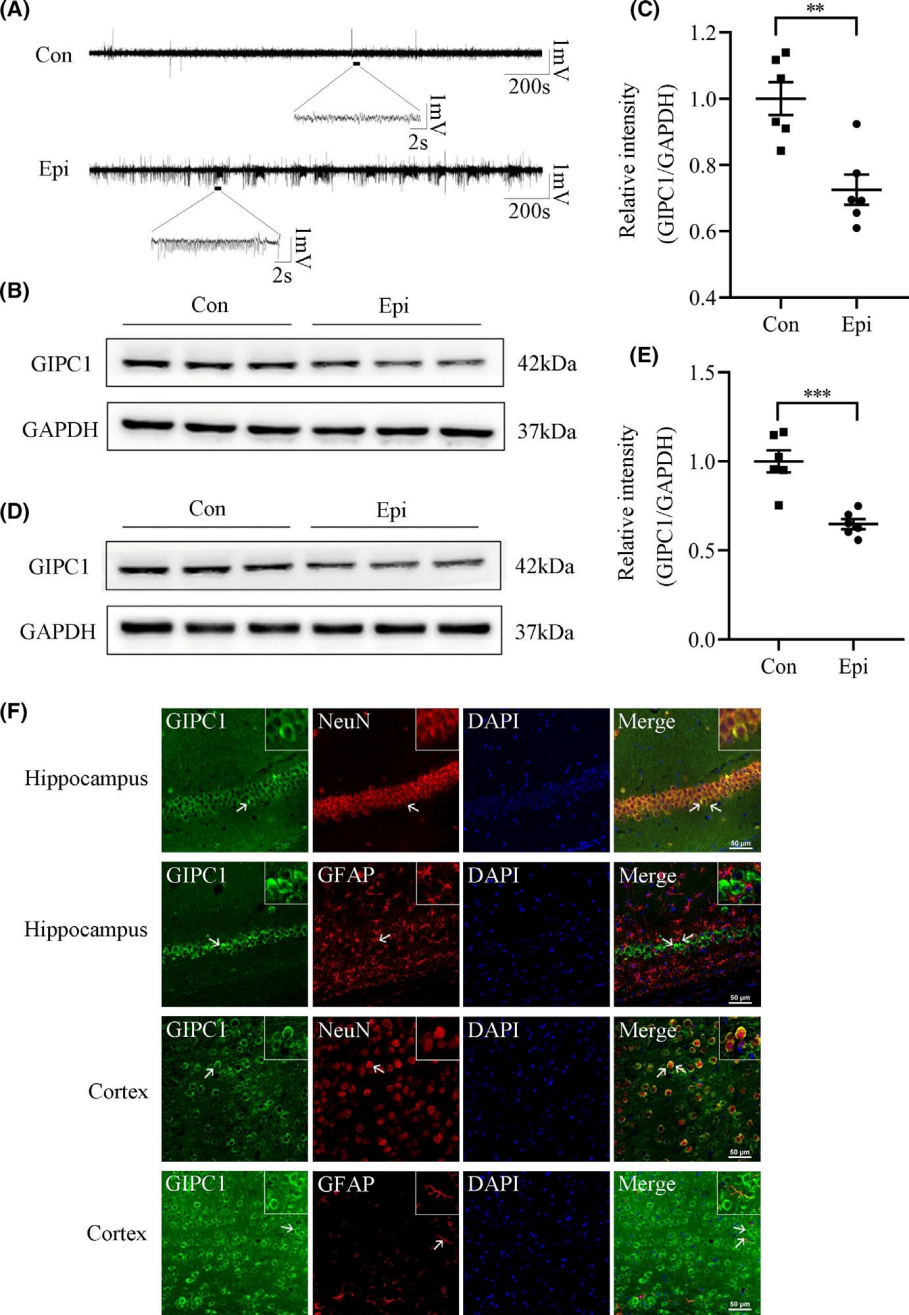
Expression and location of GAIP interacting protein, C terminus 1 (GIPC1) in the hippocampus and temporal cortex of KA‐induced epileptic mice and control mice. (A) Representative local field potential images. (B, D) Representative Western blotting images for GIPC1 in the hippocampus (B) and temporal cortex (D). (C, E) Statistical analyses showing that compared with the control group, the epilepsy group had a decreased intensity of GIPC1/GAPDH in the hippocampus (C) and temporal cortex (E) (n = 6 per group). (F) Representative double immunofluorescence images for GIPC1. GIPC1 (green) and NeuN (red) were co‐expressed (merged), while GIPC1 (green) and GFAP (red) were not co‐expressed (merged) (the upper two panels represent images of the hippocampus, and the lower two panels represent images of the temporal cortex, scale bar = 50 μm). **p < 0.01 and ***p < 0.001 [Colour figure can be viewed at wileyonlinelibrary.com]

To further investigate the relationship between GIPC1 and epilepsy, a KA‐induced epilepsy model was constructed. First, we confirmed the establishment of the model by the seizure activity of the mice and the hippocampal LFP recordings. Only mice with SRSs and epileptiform discharges were included in the epilepsy group (Figure 2A). Western blotting images for GIPC1 in the hippocampus of KA‐induced epileptic mice and control mice are shown in Figure 2B, and quantitative analysis revealed that there was a lower level of GIPC1 (*p* = 0.002; Figure 2C) in the epilepsy group than that in the control group. As revealed by immunofluorescence staining (Figure 2F, S2), GIPC1 was predominantly located in the cytoplasm of neurons but not in astrocytes, which was consistent with an earlier report.^36^ This specific distribution reveals that GIPC1 mainly exerts a regulatory effect through neurons rather than astrocytes. Furthermore, a similar expression pattern of GIPC1 was observed in the temporal cortex (*p *< 0.001; Figure 2D–F, S2).

### 3.4 AAV vector‐mediated GIPC1 expression in vivo

**FIGURE 3:**
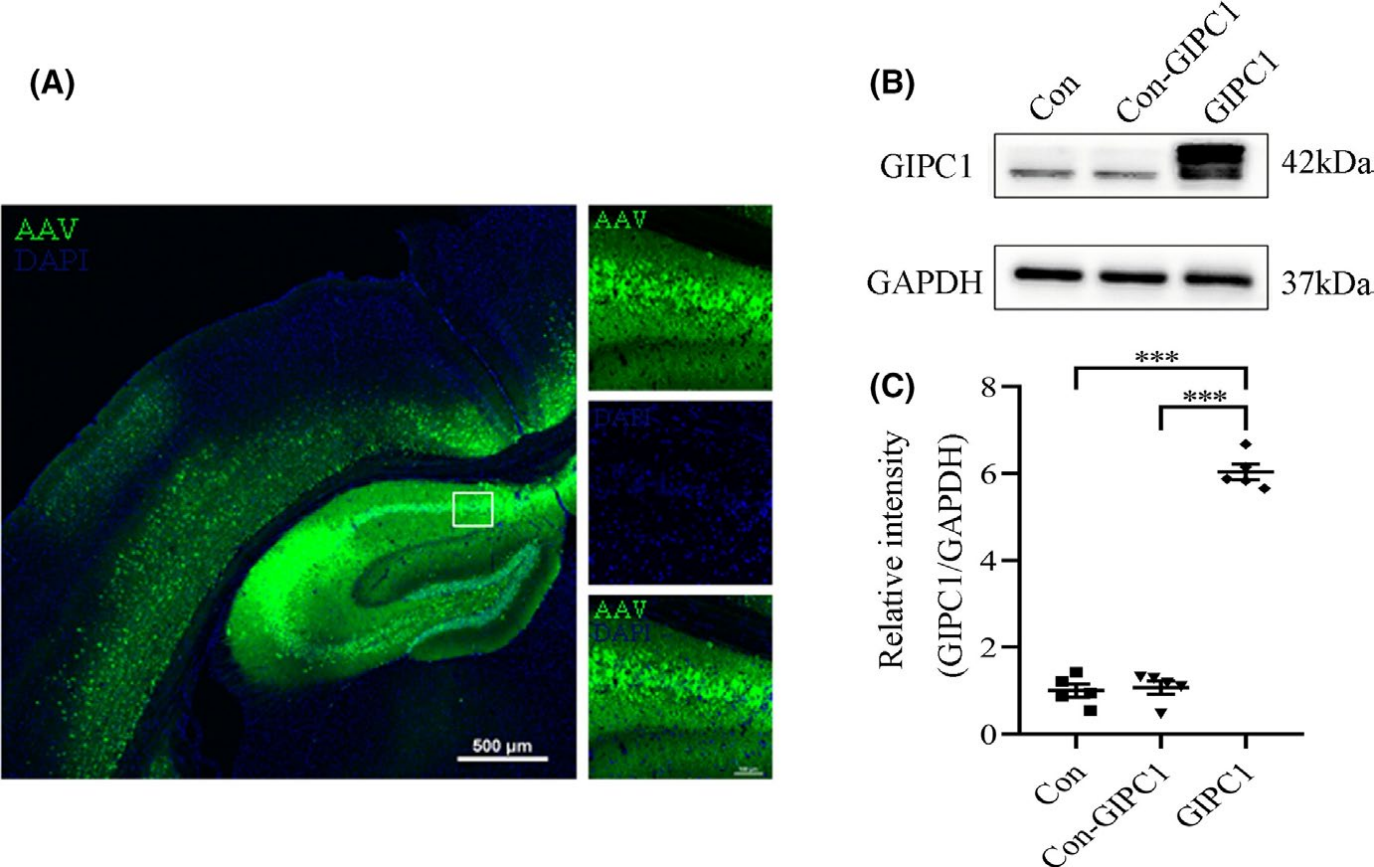
Distribution of green fluorescent protein (GFP) and expression of GAIP interacting protein, C terminus 1 (GIPC1) in the hippocampus at week 3 after injection of AAV vector. (A) Fluorescent images of the distribution of GFP (scale bar = 500 μm for the left image and scale bar = 100 μm for the right images). (B) Western blotting images for GIPC1 expression. (C) Statistical graph showing increased GIPC1 expression in the GIPC1 group compared with that in the control and Con‐GIPC1 groups (n = 5 per group). ***p < 0.001 [Colour figure can be viewed at wileyonlinelibrary.com]

To evaluate the efficiency of AAV vector‐mediated GIPC1 expression, immunostaining (Figure 3A) was performed to verify the distribution of GFP and Western blotting (Figure 3B) was performed to confirm the expression of GIPC1 at week 3 after AAV vector injection. According to Figure 3A, AAV vectors were successfully injected into the hippocampal CA1 region and widely distributed within the whole hippocampus. From Figure 3B, GIPC1 was remarkably increased in the GIPC1 group compared with that in the control and Con‐GIPC1 groups, while no obvious difference was observed between the latter two groups, which excluded interference from the AAV vector itself. The results plotted on the statistical graph (Figure 3C) led to the same conclusion (control group vs Con‐GIPC1 group: *p *= 1.000, control group vs GIPC1 group: *p *< 0.001 and Con‐GIPC1 group vs GIPC1 group: *p *< 0.001).

### 3.5 Overexpression of GIPC1 suppresses seizure susceptibility and abnormal neuronal activity in the KA‐induced chronic epilepsy model

**FIGURE 4:**
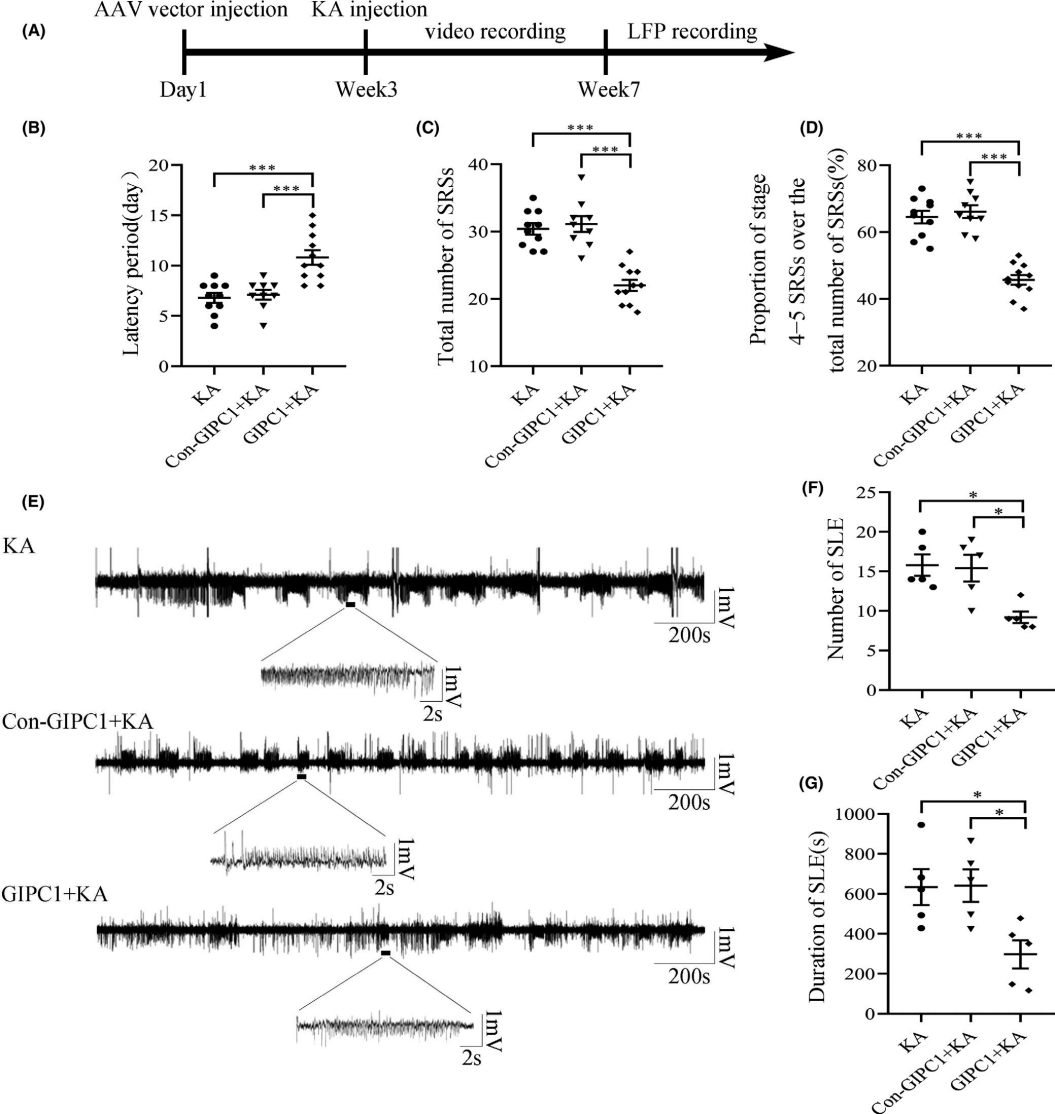
Effects of GAIP interacting protein, C terminus 1 (GIPC1) overexpression on seizure susceptibility and local field potential (LFP) in KA‐induced epileptic mice. (A) Schematic diagram of the experimental design. (B−D) Statistical graphs of the latency period (B), the total number of SRSs (C), and the proportion of stage 4−5 SRSs over the total number of SRSs (D), showing that overexpression of GIPC1 prolonged the latency, decreased the number of SRSs, and alleviated the seizure severity in the GIPC1 + KA group compared with that in the KA and Con‐GIPC1 + KA groups, respectively (n = 10 in the KA group, n = 9 in the Con‐GIPC1 + KA group, and n = 11 in the GIPC1 group). (E) Representative LFP images. (F, G) Statistical analyses indicating that both the number of SLEs (F) and the duration of SLEs (G) were decreased in the GIPC1 + KA group compared with that in the other two groups (n = 5 per group). *p < 0.05 and ***p < 0.001

Three weeks after AAV vector injection, KA was stereotactically injected into the mouse hippocampus, and then, the mice were monitored continuously for 4 weeks to evaluate their activities and seizure susceptibility (Figure 4A). According to behavioral analyses, overexpression of GIPC1 prolonged the latency period (KA group vs GIPC1 + KA group: *p *< 0.001 and Con‐GIPC1 + KA group vs GIPC1 + KA group: *p *< 0.001; Figure 4B), reduced the total number of SRSs (KA group vs GIPC1 + KA group: *p *< 0.001 and Con‐GIPC1 + KA group vs GIPC1 + KA group: *p *< 0.001; Figure 4C), and downregulated the proportion of stage 4−5 SRSs over the total number of SRSs, which represented the seizure severity (KA group vs GIPC1 + KA group: *p *< 0.001 and Con‐GIPC1 + KA group vs GIPC1 + KA group: *p *< 0.001; Figure 4D) in the GIPC1 + KA group compared with that in the KA and Con‐GIPC1 + KA groups. No significant differences in these three aspects were observed between the latter two groups (*p *= 1.000; Figure 4B; *p *= 1.000; Figure 4C; *p *= 1.000; Figure 4D).

According to LFP recordings (Figure 4E), the GIPC1 group exhibited fewer (KA group vs GIPC1 + KA group: *p *= 0.012 and Con‐GIPC1 + KA group vs GIPC1 + KA group: *p *= 0.018; Figure 4F) and shorter SLEs (KA group vs GIPC1 + KA group: *p *= 0.037 and Con‐GIPC1 + KA group vs GIPC1 + KA group: *p *= 0.033; Figure 4G) than the KA and Con‐GIPC1 + KA groups. However, the SLE number and duration were not different between the KA and Con‐GIPC1 + KA groups (*p *= 1.000; Figure 4F; *p *= 1.000; Figure 4G).

### 3.6 GIPC1 interacts with mGluR7 and overexpression of GIPC1 increases the total and cell membrane mGluR7

**FIGURE 5:**
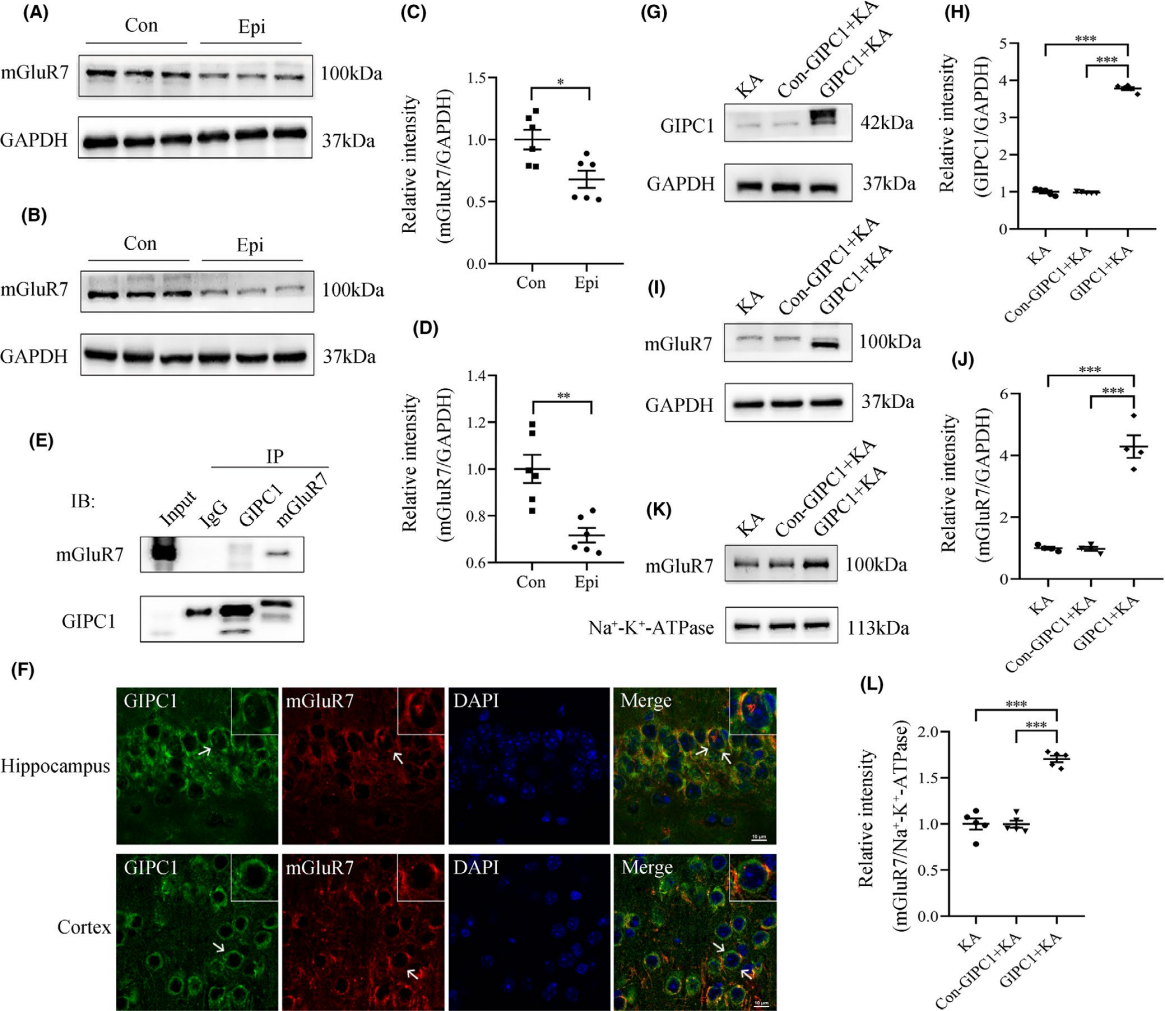
Effects of GAIP interacting protein, C terminus 1 (GIPC1) overexpression on the expression of mGluR7. (A, B) Western blotting images for mGluR7 in the hippocampus (A) and temporal cortex (B) of KA‐induced epileptic mice and control mice. (C, D) Quantitative analyses showing that in both the hippocampus (C) and the temporal cortex (D), mGluR7 was decreased in epileptic mice compared with that in control mice (n = 6 per group). (E) Co‐IP assay result of GIPC1 and mGluR7 in the hippocampus of KA‐induced epileptic mice. (F) High‐resolution immunofluorescence images for GIPC1 and mGluR7 in KA‐induced epileptic mice. GIPC1 (green) and mGluR7 (red) were co‐expressed (merged) (scale bar = 10 μm). (G, I) Western blotting images for GIPC1 (G) and mGluR7 (I) at week 4 post‐KA injection following AAV vector injection. (H, J) Statistical analyses indicating that GIPC1 was significantly increased in the GIPC1 + KA group compared with that in the KA and Con‐GIPC1 + KA groups (H); similarly, mGluR7 expression (J) showed the same difference (n = 5 per group). (K, L) Western blotting results showing that cell membrane mGluR7 in the GIPC1 + KA group was higher than that in the other two groups (n = 5 per group). *p < 0.05, **p < 0.01, and ***p < 0.001 [Colour figure can be viewed at wileyonlinelibrary.com]

As shown in Figure 5, the total mGluR7 was downregulated in the hippocampus (Figure 5A) and temporal cortex (Figure 5B) of the epilepsy group, and statistical analyses confirmed these results (*p *= 0.013; Figure 5C; *p *= 0.002; Figure 5D), which were consistent with earlier studies.^37^, ^38^ According to Figure 5E, Co‐IP assay result indicated an interaction between GIPC1 and mGluR7. In addition, high‐resolution double‐labeled immunofluorescence staining (Figure 5F and S2) also showed that GIPC1 (green) colocalized (merged) with mGluR7 (red). At week 4 post‐KA injection following AAV vector injection (7 weeks after AAV vector injection), GIPC1 expression in the GIPC1 group was much higher than that in the KA and Con‐GIPC1 + KA groups, while no evident difference between the latter two groups was observed (KA group vs Con‐GIPC1 + KA group: *p *= 1.000, KA group vs GIPC1 + KA group: *p *< 0.001 and Con‐GIPC1 + KA group vs GIPC1 + KA group: *p *< 0.001; Figure 5G, H). Following increased GIPC1 expression in the GIPC1 group, the total mGluR7 was also upregulated in the GIPC1 group, with no evident difference in the other two groups (KA group vs Con‐GIPC1 + KA group: *p *= 1.000, KA group vs GIPC1 + KA group: *p *< 0.001 and Con‐GIPC1 + KA group vs GIPC1 + KA group: *p *< 0.001; Figure 5I, J). Similar to the change of the total mGluR7, membrane mGluR7 also increased in the GIPC1 group, while no significant difference was observed between the KA and Con‐GIPC1 + KA groups (KA group vs Con‐GIPC1 + KA group: *p *= 1.000, KA group vs GIPC1 + KA group: *p *< 0.001 and Con‐GIPC1 + KA group vs GIPC1 + KA group: *p *< 0.001; Figure 5K, L).

### 3.7 Antagonism of mGluR7 increases seizure susceptibility and abnormal neuronal activity in GIPC1‐overexpressing mice

**FIGURE 6:**
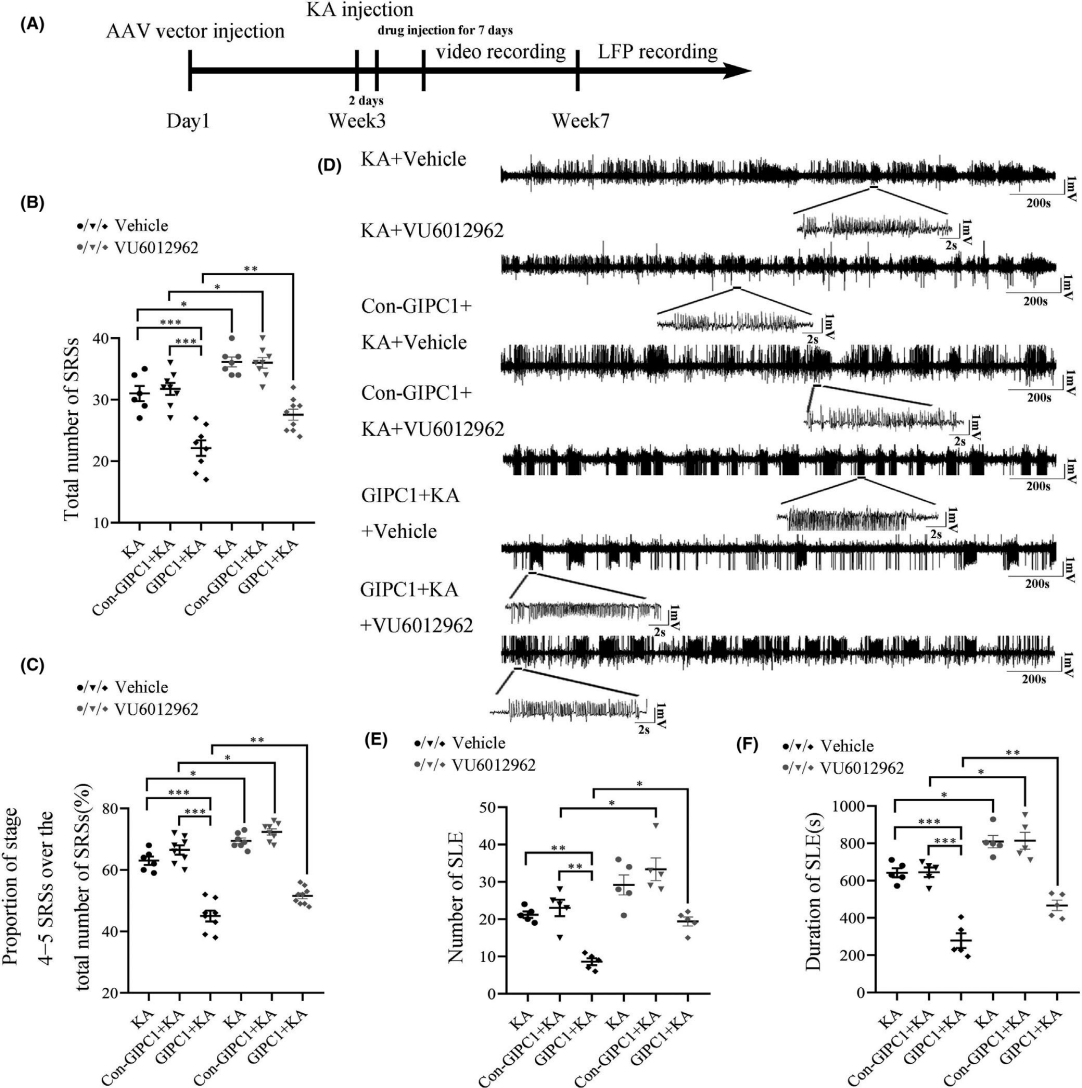
Effects of mGluR7 antagonism on seizure susceptibility and local field potential (LFP) in GAIP interacting protein, C terminus 1 (GIPC1)‐overexpressing mice. (A) Schematic diagram of the experimental design. (B, C) Statistical graphs of the total number of SRSs (B) and the proportion of stage 4−5 SRSs over the total number of SRSs (C), showing that antagonism of mGluR7 increased the total number of SRSs and aggregated the seizure severity in the VU6012962 groups compared with that in the vehicle groups (n = 6 in the KA + vehicle group, n = 8 in the Con‐GIPC1 + KA + vehicle group, n = 8 in the GIPC1 + KA + vehicle group, n = 7 in the KA + VU6012962 group, n = 8 in the Con‐GIPC1 + KA + VU6012962 group, and n = 9 in the GIPC1 + KA + VU6012962 group). (D) Representative LFP images. (E, F) Statistical analyses indicating that both the number of SLEs (E) and the duration of SLEs (F) were increased in the VU6012962 groups compared with that in the vehicle groups, except for the KA + VU6012962 group, which showed no significant difference with the KA + vehicle group in the number of SLEs (n = 5 per group). *p < 0.05, **p < 0.01, and ***p < 0.001

On the third day after KA injection following AAV vector injection, VU6012962, a highly selective NAM of mGluR7, was intraperitoneally injected into mice for 7 consecutive days (Figure 6A). According to behavioral results, antagonism of mGluR7 increased the total number of SRSs (VU6012962 vs vehicle: KA: *p* = 0.04, Con‐GIPC1 + KA: *p* = 0.04 and GIPC1 + KA: *p* = 0.008; Figure 6B) and upregulated the proportion of stage 4−5 SRSs over the total number of SRSs (VU6012962 vs vehicle: KA: *p* = 0.038, Con‐GIPC1 + KA: *p* = 0.032 and GIPC1 + KA: *p* = 0.005; Figure 6C) in the VU6012962 groups compared with that in the vehicle groups. While among the vehicle groups, the GIPC1 + KA + vehicle group showed fewer number of SRSs and lower proportion of stage 4−5 SRSs over the total number of SRSs than the KA + vehicle and Con‐GIPC1 + KA + vehicle groups. No significant differences were observed in these two aspects between the latter two groups, which was consistent with the results in Figure 4C and 4D (Exact *p*‐values are shown in Table S1). To further exploration, we carried out LFP recordings (Figure 6D). Similarly, the VU6012962 groups exhibited increased number of SLEs (VU6012962 vs vehicle: Con‐GIPC1 + KA: *p* = 0.018 and GIPC1 + KA: *p* = 0.013; Figure 6E) and increased duration of SLEs (VU6012962 vs vehicle: KA: *p* = 0.027, Con‐GIPC1 + KA: *p* = 0.024 and GIPC1 + KA: *p* = 0.008; Figure 6F) in comparison with the vehicle groups. On the other hand, the KA + VU6012962 and KA + vehicle groups had no significant difference in the number of SLEs (*p* = 0.142; Figure 6E). Additionally, among the vehicle groups, the GIPC1 + KA + vehicle group showed fewer and shorter SLEs than the KA + vehicle and Con‐GIPC1 + KA + vehicle groups with no significant differences observed in these two aspects between the latter two groups, which was consistent with the results in Figure 4F and 4G (Exact *p*‐values are shown in Table S1).

## 4 DISCUSSION

In this study, we preliminarily described the role of GIPC1 in epileptogenesis and drew three conclusions. First, compared with that in the control group, the expression of GIPC1 decreased in the brain tissues of patients with TLE and mice with KA‐induced epilepsy. Second, further studies showed that elevated GIPC1 lowered seizure susceptibility and reduced epileptic activity in behavioral tests and decreased abnormal discharges in LFP recordings. Third, GIPC1 interacted with mGluR7, and after GIPC1 overexpression, the total and cell surface mGluR7 levels increased as well. In addition, inhibition of mGluR7 partially reversed the antiepileptic effect of GIPC1.

Epilepsy is a common neurological disease characterized by recurrent seizures. Numerous studies have shown that abnormal synaptic transmission is an important pathogenetic mechanism.^8^, ^9^, ^10^ As a scaffolding protein, GIPC1 is relatively highly expressed in various normal tissues including brain tissue.^12^ Since GIPC1 regulates the cell surface expression and endocytic trafficking of numerous transmembrane receptors and signaling complexes,^39^ it plays an important role in synaptic transmission. To date, it has been reported that there are more than 50 protein‐binding partners of GIPC1, most of which play essential roles in neuronal development,^13^ which suggests that GIPC1 may play a multifaceted role in the CNS and may also be involved in epilepsy. Thus, we explored the expression patterns of GIPC1 in the brain tissues of patients with TLE and mice with KA‐induced epilepsy and our experimental results of reduced GIPC1 also show the possible involvement of GIPC1 in epilepsy. In addition, it has been reported that gene mutations in the GIPC protein family may be associated with the development of myoclonus, primary tremor, and juvenile epilepsy,^14^ and previous studies have indicated that many scaffolding proteins^15^, ^16^, ^17^ are involved in epilepsy; hence, we speculate that GIPC1 also participates in epilepsy. Thus, we conducted behavioral tests and LFP recordings to identify the role of GIPC1 in epileptogenesis. The results in the present study provide strong evidences to support this hypothesis and also show an antiepileptic protective effect of GIPC1.

Due to the important role of synaptic transmission in epileptogenesis and the important role of GIPC1 in synaptic transmission, we speculate that GIPC1 is involved in epileptogenesis by regulating synaptic transmission. mGluR7, a metabotropic glutamate receptor, is predominantly located on the presynaptic membrane, and it serves as an autoreceptor suppressing the release of glutamate from the presynapses, thus acting as a negative modulator of epilepsy.^40^, ^41^ Interestingly, there is a PBM in mGluR7, which means that it can interact with PDZ proteins. PICK1, similar to GIPC1, is a PDZ scaffolding protein. Previous researches have shown that PICK1 can bind to the PBM of mGluR7 and then stabilize and increase the cell surface expression of mGluR7 together with PKC.^40^, ^42^ Meanwhile, researchers have shown that PICK1 uncoupling from mGluR7a causes absence‐like seizures.^43^ Therefore, we speculate that GIPC1, like PICK1, can bind to mGluR7 via its PDZ domain and increase cell surface mGluR7 expression and then inhibit the release of glutamate from presynapses, thus exerting an antiepileptic effect. The positive result in the Co‐IP assay and the colocalization of GIPC1 and mGluR7 in the high‐resolution fluorescence staining verify the interaction between these two proteins. As part of our findings which showed that an increased mGluR7 expression followed the increased GIPC1, we proved the involvement of mGluR7 in the GIPC1‐mediated epileptogenesis mechanism by antagonism of mGluR7. Thus, the hypothesis mentioned above was confirmed. Although a positive result was observed in the Co‐IP assay, the molecular weights of GIPC1 and mGluR7 increased, which may be the results of post‐translational modification.^40^, ^44^, ^45^ The modified GIPC1 band (above 42 kDa) in the Input and GIPC1 lane is shallower than the unmodified GIPC1 band (42 kDa), and the darkest band on the top is the IgG heavy chain, suggesting that GIPC1 mainly exists in the unmodified state in the hippocampus of the KA‐induced epileptic mice and may mainly act through the modified state. Only the modified GIPC1 band is observed in the mGluR7 lane, suggesting that it mainly binds to the modified GIPC1. Because the IgG heavy chain band is too dark, resulting in a poorly imaged immunoprecipitation band, we overexposed this band to obtain a more obvious immunoprecipitation band (Figure S1).

In addition, there are some limitations in this study that need to be acknowledged. First, we used sodium pentobarbital to anesthetize mice. Previous studies have identified the antiepileptic effect of sodium pentobarbital, which is often used to treat refractory and super‐refractory status epilepticus by continuous intravenous infusion.^46^, ^47^ However, we anesthetized mice with a single, small intraperitoneal injection before KA injection, and SRSs representing epilepsy formation only appeared in the chronic phase. Furthermore, sodium pentobarbital, a commonly used anesthetic, is often found during the construction of animal models of epilepsy.^48^, ^49^ Second, GIPC1 expression was not detected in the hippocampus of TLE patients and control subjects for practical and ethical reasons. Although the expression patterns of GIPC1 in the temporal neocortex of TLE patients are less persuasive than those in the hippocampus, they also reflect the change of GIPC1 in the brain of patients to a certain extent. Third, what downstream signaling mediated by mGluR7 to play a role in the regulatory mechanism of GIPC1 and the mechanism mediated by GIPC1 to increase the level of mGluR7 remain unknown, which needs to be further investigated in the future.

In conclusion, GIPC1 is downregulated in the brain tissues of patients with TLE and mice with KA‐induced epilepsy. Overexpression of GIPC1 lowers seizure susceptibility and decreases abnormal neuronal discharges. In addition, GIPC1 interacts with mGluR7. Along with GIPC1 overexpression, the total and cell surface expression levels of mGluR7 are also overexpressed, and antagonism of mGluR7 partially reverses the antiepileptic effect of GIPC1. This proves the involvement of mGluR7 in the GIPC1‐mediated epileptogenesis mechanism and makes GIPC1 a promising therapeutic target for epilepsy.

## CONFLICT OF INTEREST

There is no conflict of interest.

## AUTHOR CONTRIBUTIONS

YL designed and performed the study, analyzed the data, and wrote the manuscript. YW designed the study, reviewed, and edited the manuscript. JY conceived the study and designed the methodology. TX designed the methodology, reviewed, and edited the manuscript. CT and PZ conceived the study and designed the methodology. QL performed the study. YC conceived and supervised the study, administered the project, and was involved in funding acquisition. All authors have read and approved the final manuscript.

## Supporting information

Fig S1

Fig S2

Table S1

## Data Availability

The data that support the findings of this study are available from the corresponding author upon reasonable request.
