# Supplementary figures and images for: G‐alpha interacting protein interacting protein, C terminus 1 regulates epileptogenesis by increasing the expression of metabotropic glutamate receptor 7
Source: CNS Neurosci Ther. 2021 Oct 22;28(1):126–38. doi: 10.1111/cns.13746 (PMC8673704; doi:10.1111/cns.13746)

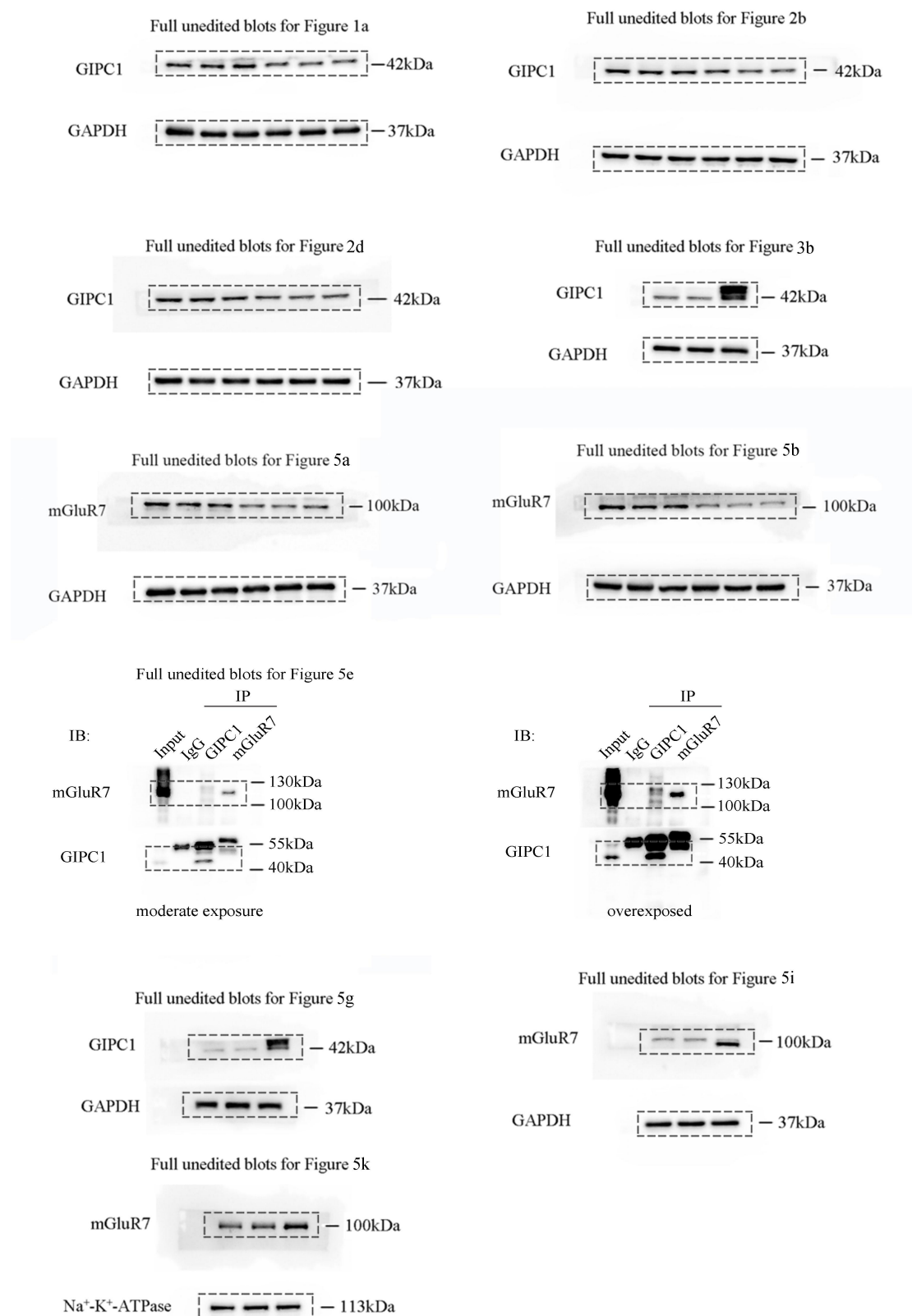

Figure S1. Uncropped images of Western blots.

Supplement: Supplementary file 1 — Fig S1 [file CNS-28-126-s001.pdf]
