# Supplementary material for: G‐alpha interacting protein interacting protein, C terminus 1 regulates epileptogenesis by increasing the expression of metabotropic glutamate receptor 7
Source: CNS Neurosci Ther. 2021 Oct 22;28(1):126–38. doi: 10.1111/cns.13746 (PMC8673704; doi:10.1111/cns.13746)
Supplement: Supplementary file 2 — Fig S2 [file CNS-28-126-s003.doc]

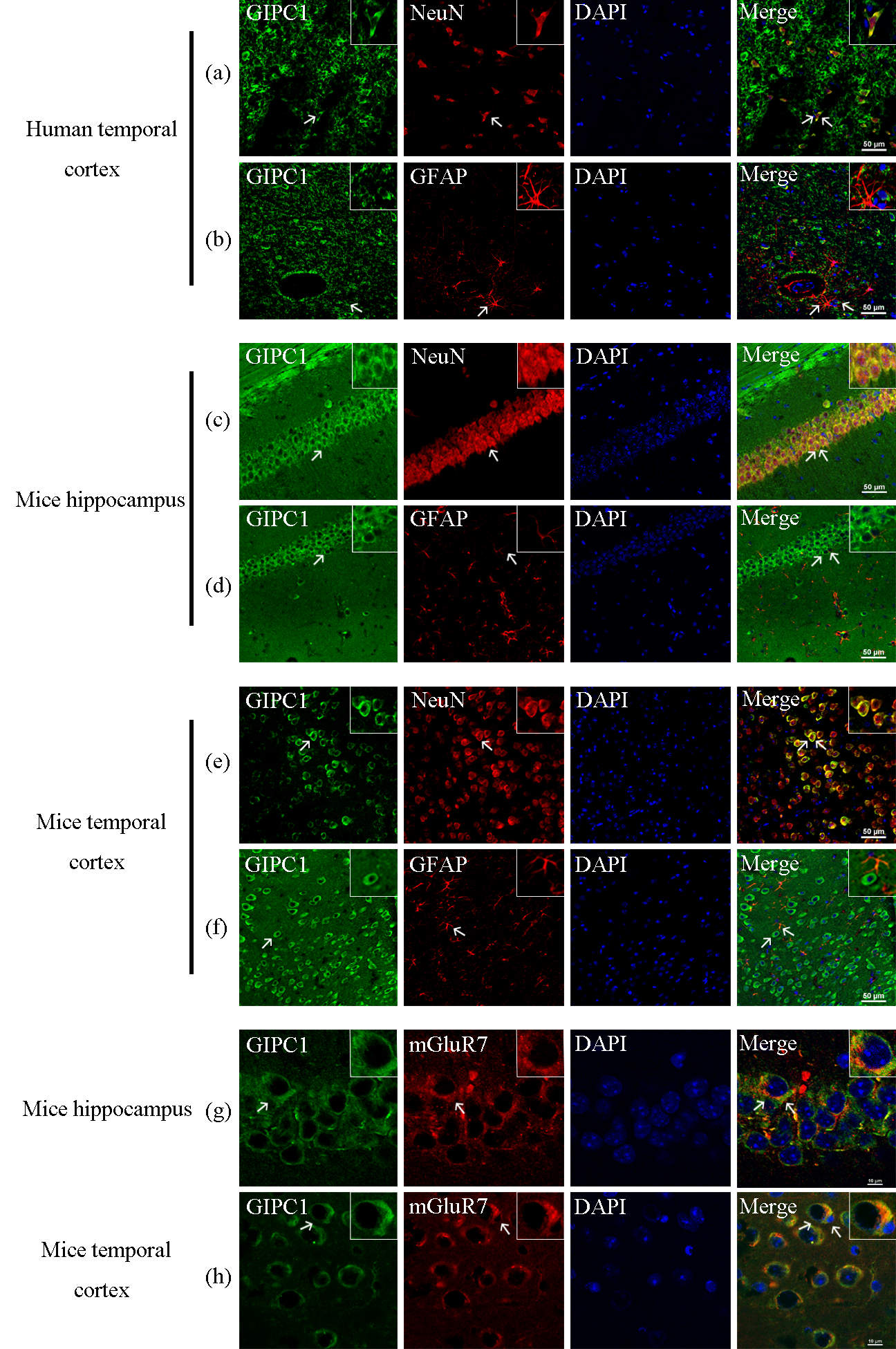


Figure S2. Double immunofluorescence images of control subjects and control mice. (a, b) Representative double immunofluorescence images for GIPC1 in the temporal cortex of control subjects. GIPC1 (green) co-expressed (merged) with NeuN (red) but not with GFAP (red) (scale bar = 50 µm). (c−f) Representative immunofluorescence images for GIPC1 in the hippocampus (c, d) and temporal cortex (e, f) of control mice. GIPC1 (green) co-expressed (merged) with NeuN (red) but not with GFAP (red) (scale bar = 50 µm). (g, h) High-resolution immunofluorescence images for GIPC1 and mGluR7 in the hippocampus (g) and temporal cortex (h) of control mice. GIPC1 (green) and mGluR7 (red) were co-expressed (merged) (scale bar = 10 µm).
