# Supplementary material for: G‐alpha interacting protein interacting protein, C terminus 1 regulates epileptogenesis by increasing the expression of metabotropic glutamate receptor 7
Source: CNS Neurosci Ther. 2021 Oct 22;28(1):126–38. doi: 10.1111/cns.13746 (PMC8673704; doi:10.1111/cns.13746)
Supplement: Supplementary file 3 — Table S1 [file CNS-28-126-s002.doc]

Table S1 Exact p-values of Figure 6 (b, c, e and f) among the vehicle groups in main text.

| p-values | p-value of the total number of SRSs | p-value of the proportion of stage 4−5 SRSs over the total number of SRSs | p-value of the number of SLEs | p-value of the duration of SLEs |
| --- | --- | --- | --- | --- |
| KA+vehicle group vs Con-GIPC1+KA+vehicle group | 1.000 | 1.000 | 1.000 | 1.000 |
| KA+vehicle group vs GIPC1+KA+vehicle group | < 0.001 | < 0.001 | = 0.003 | < 0.001 |
| Con-GIPC1+KA+vehicle group vs GIPC1+KA+vehicle group | < 0.001 | < 0.001 | = 0.001 | < 0.001 |
